# Fetal monitoring—a risky business for the unborn and for clinicians

**DOI:** 10.1111/j.1471-0528.2008.01758.x

**Published:** 2008-07

**Authors:** I Amer-Wahlin, S Dekker

**Affiliations:** a Department of Women and Child Health, Karolinska University HospitalStockholm, Sweden; b Lund University School of AviationLjungbyhed, Sweden

In their recent report on three deliveries with adverse outcomes, Westerhuis *et al.*^1^ describe the clinical application of the ST-analysis of fetal EG technology as an additional source of information in fetal monitoring. The title of their article and the conclusion imply that the method contributed to the adverse outcomes. However, closer reading gives quite a different picture. In relation to case 1 the authors say:…. ‘… guidelines were not followed because they indicate immediate delivery in the case of a (pre)terminal CTG. This case illustrates the difficulty of classification of the CTG…’ (p. 1199). About case 2 they say ‘A preterminal CTG pattern… which should have been acted upon at an earlier stage…’ (p. 1200) and about case 3: ‘Perhaps the most important lesson from this case is that one should continue to assess the CTG’ (p. 1200).

What is described in the three cases is thus either a situation with preterminal cardiotocogram (CTG) where no action was taken or misinterpretation of CTG and/or the staff passively awaiting an ST event.

The authors’ conclusion should come as no surprise: ‘The most important limitation of ST analysis is deviation from STAN clinical guidelines by labor ward personnel rather than a fault in the technology’ (p. 1199).

Who then should be blamed? The monitoring technology? The guidelines? The obstetrician? The midwife? What are the reasons for inaction when action is called for and when both the technology and the guidelines recommend action? With hindsight it all seems illogical, and anyone who was not there finds it difficult to understand what happened. We fail to appreciate that difficult trade-offs are necessary when the clinicians have to make their decisions in a busy, uncertain and ‘noisy’ setting. The easiest way out is to blame technology or the human involved. However, it is more interesting and productive to start asking why, instead of who. Accidents or adverse events are seldom the result of one single mistake, but emerge from a host of factors.

How can we explain why the personnel did not apply the methods of CTG interpretation that they and their senior colleagues have practised since CTG was introduced in the 1960s. Why does the arrival of a technical device adding ST analysis to CTG, make us behave like the onlookers who saw their first automobile? It is likely, of course, that problems with CTG interpretation have always existed and that a balanced, evidence-based assessment of its risks has not been able to compete with our tendency to stick with established customary practices. One wonders whether our resistance to alternative technologies and practices and our overconfidence in the effectiveness of traditional techniques (‘this is how we’ve always done it’) may perhaps benefit powerful regulatory, academic and industry interests at the heart of the development of medical technology, which tend to inhibit innovation.

Comparing what is going on in the delivery room to another risky activity such as flying immediately reveals the huge difference between the amount of attention and resources put into the two. A whole science has developed around risk and safety in aviation and it pays off. It is obvious that adverse outcomes related to errors occur far more often in the delivery rooms than in the air. Why is it so? Risk and safety are not areas of high priority in the delivery room even if risk analysis and mitigation systems have been successfully and systematically introduced in various local settings, for example, in the UK (http://www.msnpsa.nhs.uk). Four decades of mistakes linked to misinterpretation in the field of fetal monitoring with CTG have not even provided us with safe procedures and a system memory of previous errors.

In a commentary linked to the article, Ingemarsson and Westgren^2^ describe 12 Swedish cases from a 4-year period illustrating what they term ‘false-negativity’, caused by ST analysis, as an adjunct to CTG. Again the underlying message is that technology has indirectly caused the adverse outcomes. Investigations undertaken by the Swedish Board of Health and Welfare in some of the cases have shown that the adverse outcomes were caused by the staff not taking action upon abnormal CTG patterns, misinterpretation of the CTG and/or incorrect use of STAN because of lack of proper training. In the same period during which Ingemarsson and Westgren report their 12 cases, many more cases with adverse outcomes related to the use of CTG alone occurred in Sweden. A recent Swedish study^3^ describes CTG misinterpretation as the main issue in relation to ‘malpractice’. The authors conclude that fetal surveillance and attention to signs of asphyxia must be improved. The situation is probably the same in the Netherlands from which Westerhuis *et al.* reported the three case studies.

Electronic fetal monitoring (CTG) was introduced in the 1960s with the aim of decreasing perinatal mortality and morbidity. The expected benefits have only partly been obtained and medico-legally, obstetrics has during the same time period become both more dangerous and more expensive for the professional. Defensive or even pre-emptive intervention to avoid negligence claims is a reality. This is not surprising and might even be acceptable if it actually produced an effect on neonatal morbidity. But such an effect has not been observed and cases of delayed intervention or non-intervention with negative outcome obviously still occur. CTG misinterpretation is one of the great risks in the delivery room.^4^,^5^ Not striving to improve the situation through additional or new methods is, of course, unethical. ST analysis of fetal ECG is such an improvement. CTG will always be a nonspecific method, currently dependent heavily on subjective interpretation. Thus, the personnel (and the fetus) remain at risk for wrong/delayed action as clear-cut information is not available. Only with the addition of nonsubjective information will the risk decrease. Results from the clinical use of automatic ST analysis is emerging, confirming that if used according to guidelines, CTG with the addition of ST analysis of fetal ECG is superior to CTG alone.^6^–^8^ However, the technology needs further development such as online computer analysis to help avoid misinterpretation of CTGs; and, as with any other field that engages high-technology devices to support safety-critical work, the need for regular training and systematic proficiency checking cannot be overemphasised. It should not be seen as a problem of resources, but perhaps one of regulation: a possible scenario is, for example, that no maternity unit would be allowed to operate such technology without a validated programme of initial staff training and regular proficiency checks. Submitting to such scrutiny appears much less of a problem in, for example, aviation, where an entire professional career consists of a string of checks, reviews, tests and more checks.

Adverse outcomes should drive exploration, reflection, development and improvement, not retribution and finger-pointing. The intensive study of organisational accidents over the past 30 years consistently shows that attributing an adverse event to a narrow proximal cause (technological failure, human error) may give an illusion of understanding, but produces only sterile responses.^9^

The underlying but misguided idea is often that ‘human error’ (by any other label: mis-assessment, misdiagnosis, misuse of an otherwise flawless technology) is a satisfactory explanation of failure. In contrast to this idea, we believe that ‘human error’ is something that *demands* an explanation. Human error can never be the conclusion of an investigation into an adverse event. Instead, it should be the starting point. We should not see human error as the cause; but as a symptom, an expression, of inadequacies deeper within the organisational, technological and operational system that makes up clinical work. If we really want to find out what goes wrong and how to improve it, we need to go beyond that first, apparently simple story that holds technological failure or human error responsible. When we do such deeper investigations, we discover much more complex patterns of clinical practice and technological support that in some cases make people excel and in other situations undermines their expertise. It is in this underlying story that we can begin to discern how clinicians handle difficult situations under immense uncertainty and ever-present time pressure, where different technologies offer different mixes of capabilities and complexities. It is here that we can begin to see how practitioners cope with the complexities of their actual work (including new technology), and from this we can learn. This is what civil and military aviation have done.^10^ The call is for digging deeper, in order to understand why, not to disperse guilt. Traditionally, reactions to failure assume that safety gets undermined by unreliable technology or by the unpredictable, erratic assessments of human beings. But instead, studies of clinicians and other safety-critical professions show how people routinely create safety through practice by attuning and enhancing their awareness of hazards and adapting their practices and technologies to guard against or defuse threats to safety. Things go wrong when people's ability to adapt successfully is weakened, for example, by time constraints, inadequate training or goal conflicts and things will likely keep going wrong if we rely on facile, superficial explanations for those failures. The greatest risk to safety in the delivery room is not the technology, nor the human. It is oversimplification: the idea that there are simple explanations for adverse events and single silver bullets that can resolve the situation is an illusion. Our patients deserve deeper, more complex explanations that take account of human behaviour—and can lead to real improvements in practice.
